# Choline supplementation prevents diet induced gut mucosa lipid accumulation in post-smolt Atlantic salmon (*Salmo salar* L.)

**DOI:** 10.1186/s12917-020-2252-7

**Published:** 2020-01-31

**Authors:** Anne Kristine G. Hansen, Trond M. Kortner, Aleksei Krasnov, Ingemar Björkhem, Michael Penn, Åshild Krogdahl

**Affiliations:** 1 Biomar AS, Havnegata 9, 7010 Trondheim, Norway; 2 Department of Paraclinical Sciences, Faculty of Veterinary Medicine, Norwegian University of Life Sciences, Oslo, Norway; 3 Nofima AS, Ås, Norway; 4 Department of Laboratory Medicine, Division for Clinical Chemistry, Karolinska University Hospital, Huddinge, Sweden; 5 Present Address: US Fish & Wildlife Service, Northeast Fishery Center, Lamar Fish Health Center, Lamar, PA 16848 USA

**Keywords:** Choline, Lipid accumulation, Lipid transport, LMS, Lipid malabsorption, Gut health, Fish feed, Plant ingredients

## Abstract

**Background:**

Various intestinal morphological alterations have been reported in cultured fish fed diets with high contents of plant ingredients. Since 2000, salmon farmers have reported symptoms indicating an intestinal problem, which we suggest calling lipid malabsorption syndrome (LMS), characterized by pale and foamy appearance of the enterocytes of the pyloric caeca, the result of lipid accumulation. The objective of the present study was to investigate if insufficient dietary choline may be a key component in development of the LMS.

**Results:**

The results showed that Atlantic salmon (*Salmo salar*), average weight 362 g, fed a plant based diet for 79 days developed signs of LMS. In fish fed a similar diet supplemented with 0.4% choline chloride no signs of LMS were seen. The relative weight of the pyloric caeca was 40% lower, reflecting 65% less triacylglycerol content and histologically normal gut mucosa. Choline supplementation further increased specific fish growth by 18%. The concomitant alterations in intestinal gene expression related to phosphatidylcholine synthesis (*chk* and *pcyt1a*), cholesterol transport (*abcg5* and *npc1l1*), lipid metabolism and transport (*mgat2a* and *fabp2*) and lipoprotein formation (*apoA1* and *apoAIV*) confirmed the importance of choline in lipid turnover in the intestine and its ability to prevent LMS. Another important observation was the apparent correlation between *plin2* expression and degree of enterocyte hyper-vacuolation observed in the current study, which suggests that *plin2* may serve as a marker for intestinal lipid accumulation and steatosis in fish. Future research should be conducted to strengthen the knowledge of choline’s critical role in lipid transport, phospholipid synthesis and lipoprotein secretion to improve formulations of plant based diets for larger fish and to prevent LMS.

**Conclusions:**

Choline prevents excessive lipid accumulation in the proximal intestine and is essential for Atlantic salmon in seawater.

## Background

The main driver for replacement of marine raw materials with alternative plant ingredients in fish feed is the ambition to maintain growth of the aquaculture industry and to secure flexibility regarding raw materials in feed production. However, in parallel with the decrease in fishmeal and increase in plant meals in fish feed, the prevalence of various intestinal disturbances has increased. Therefore, it is likely that some of the observed intestinal challenges may be due to deficient supply of nutrients, which are present at lower levels in plant ingredients than in fishmeal, but not corrected for due to lack of information on their essentiality and/or required level. The requirements for many nutrients have been defined for many species but all nutrient requirements are far from defined [1].

The present work addresses symptoms of a well-known intestinal disorder for which we suggest the term lipid malabsorption syndrome (LMS) and which since 2000 have been reported by salmon farmers to affect young as well as more mature fish [2–4]. The typical sign is increased lipid accumulation in the enterocytes giving the pyloric caeca a pale and foamy appearance on the macroscopic level. Similar signs have been reported also in other fish species fed diets high in plant meal [5–8] or high in plant oil [7, 9–11]. The apparent disturbance in lipid transport is also observed on the molecular level. Plant based diets may influence the expression of genes involved in lipid metabolism in a manner reflecting reduced lipid export from the enterocytes [8, 12–20]. However, the mechanisms underlying the excessive lipid accumulation are not yet fully clarified. Some studies seem to indicate that phospholipid synthesis, and in particular phosphatidylcholine, might be the bottleneck in lipid export from the enterocytes in fish showing such lipid accumulation [9, 10, 21–27]. Phosphatidylcholine, however, is not established as an essential nutrient for Atlantic salmon nor for any other fish species (NRC, 2011). For choline, on the other hand, of which about 95% is found in phosphatidylcholine [28, 29], a requirement is established for several fish [1]. Due to insufficient information, the question of whether choline is essential, and if so, the required amount, cannot currently be determined for Atlantic salmon.

In animals, including fish, choline is necessary for synthesis of phosphatidylcholine for use in lipid digestion and absorption, as a component in lipoproteins for lipid transport, in production of the neurotransmitter acetylcholine, and as a methyl donor in a wide range of methylation processes. Poor growth and low feed efficiency, fatty liver, high mortality, and anorexia are all reported effects of choline deficiency in the species for which we have documentation [1, 30–32]. Lipid accumulation in the intestinal mucosa is, however, not a common endpoint in studies of choline deficiency and requirement and has only been observed in an early study of Japanese eel (*Anguilla japonica*) as “white-grey colored intestines” [33]. This gut observation appears similar to that observed in Atlantic salmon with LMS. No studies have been conducted to define whether choline is essential for Atlantic salmon, or how much can be synthesized. Accordingly, a requirement is not established, and the question whether high plant diets might be deficient in phosphatidylcholine or choline, cannot be answered. Rainbow trout have been found to require choline at earlier life stages due to an inability to produce sufficient choline even with a high supply of methyl donors such as betaine and methionine [31]. On the other hand, channel catfish were able to produce sufficient choline, if the supply of methionine was high [1].

The level of fishmeal in today’s commercial salmon diets is in general low and decreases throughout the life cycle of the fish. A diet for salmon weighing 500 g or more typically contains between 5 and 10% fishmeal. Fishmeal would be the main contributor for choline in these diets, which means that supply of choline from the other main ingredients would be rather low. For example, the basal diet (LF) used in the present experiment was a commercially representative feed with 10% fishmeal which revealed a choline level of 944 mg/kg (Tables 1 and 2). With several recent reports from the salmon industry regarding LMS [4], investigation of the role of choline for LMS is needed. The present work therefore aimed to elucidate whether LMS is a result of insufficient choline supply and also addressed the role of choline in enterocyte lipid transport in post-smolt Atlantic salmon.

**Table 1:** Formulation and chemical composition of the experimental diets

Diets	LF ^a^	LFC ^b^
Ingredients (g/kg)		
Super Prime fra Peru^c^	50	50
Nordic LT 94 fishmeal ^d^	50	50
Soya 60% (SPC) ^e^	190	194
Maize Gluten ^f^	150	150
Pea Protein 50 ^g^	130	130
Dehulled Beans ^h^	140	130
Wheat Gluten ^i^	19.7	19.7
Fish oil (Standard) ^j^	76.7	77.1
Rapeseed oil ^k^	176	177
Amino Acid mix ^l^	12.4	12.4
Mineral mix ^l^	3.0	3.0
Monocalcium phosphate ^l^	18.2	18.2
Lucantin Pink CWD 10% ^l^	0.4	0.4
Yttrium ^m^	0.5	0.5
Choline chloride 70%	0	4.0
Analyzed chemical composition (g/kg)		
DM	975	972
Protein	417	418
Fat	286	297
Starch	107	102
Total choline (mg/kg)	944	4250
Total methionine	9.1	9.4
Total cysteine	5.1	5.8

^a^Low fishmeal diet

^b^Choline supplemented low fishmeal diet

^c^Supplied by Kôster Marine Proteins GmbH

^d^Supplied by Norsildmel AS

^e^Supplied by Selecta S/A, Avenida Jamel Ceilio, 2496 – 12th region. SPC, soya protein concentrate

^f^Supplied by Cargill Nordic

^g^Supplied by DLG Food Grain

^h^Supplied by HC Handelscenter

^i^Supplied by Roquette

^j^Supplied by FF Skagen

^k^Supplied by Emmelev

^l^Supplemented to meet the requirements

^m^Inert marker for the evaluation of nutrient digestibility

**Table 2:** Growth performance (Mean values with their standard errors)

	LF^a^	LFC^b^	Pooled SEM	*P*-value^c^
IBW (g)^d^	364	354	7.3	0.501
Growth (g)	344	418	20.3	**< 0.001**
SGR (%d-1)^e^	0.84	0.99	0.04	**< 0.001**

^a^Control low fishmeal diet group (*n* = 70)

^b^Choline supplemented low fishmeal diet (*n* = 68)

^c^*P*-values obtained in t-test, values in bold indicate significant differences between the two treatments

^d^Initial body weight

^e^Specific growth rate

## Results

### Growth performance and nutrient digestibilities

Growth performance was significantly higher for fish fed the choline supplemented feed (LFC) compared to those fed the unsupplemented basal diet (LF, Table 2). Choline inclusion did not affect apparent digestibility (AD) significantly for any of the nutrients. The average AD (± SEM) for the two test diets was 96.1 (± 0.24) for crude lipid, 90.1 (± 0.19) for crude protein and 75.8 (± 0.65) for starch.

### Intestinal chyme dry matter and brush border leucine aminopeptidase

Choline supplementation tended to increase dry matter content of digesta along the intestine (Table 3). The increase was significant for the mid intestine (MI) and distal intestine (DI) sections of the intestine. The trend was clear also for proximal half of the pyloric intstine 1 (PI1) and PI2 (*p* = 0.062 and 0.086, respectively). Brush border membrane leucine aminopeptidase (LAP) activities for PI and DI are shown in Table 3. There were no significant differences in the enzyme activity between the two treatments either in PI or DI tissue.

**Table 3:** Intestinal dry matter and leucine aminopeptidase activity (LAP) (Mean values with their standard errors)

	LF^a^	LFC^b^	Pooled SEM	*P*-value^c^
*Intestinal dry matter (%)*				
PI1	9.3	10.3	0.65	*0.062*
PI2	11.0	12.2	0.71	*0.086*
MI	12.9	14.5	0.67	**0.005**
DI1	12.9	14.0	0.48	**0.011**
DI2	11.0	12.7	0.47	**<0.001**
*LAP (mmol/h/kg BW)*				
PI	244	235	16.1	0.729
DI	44	43	3.1	0.743
*LAP (μmol/h/mg prot)*				
PI	331	385	25.3	0.309
DI	228	244	17.3	0.237

^a^Control low fishmeal diet group (*n* = 20)

^b^Choline supplemented low fishmeal diet (*n* = 20)

^c^*P*-values in bold indicate significant differences between the two treatments; italicized values represent trends

### Organosomatic indices, intestinal and liver lipid content and histology

**Fig. 1:**
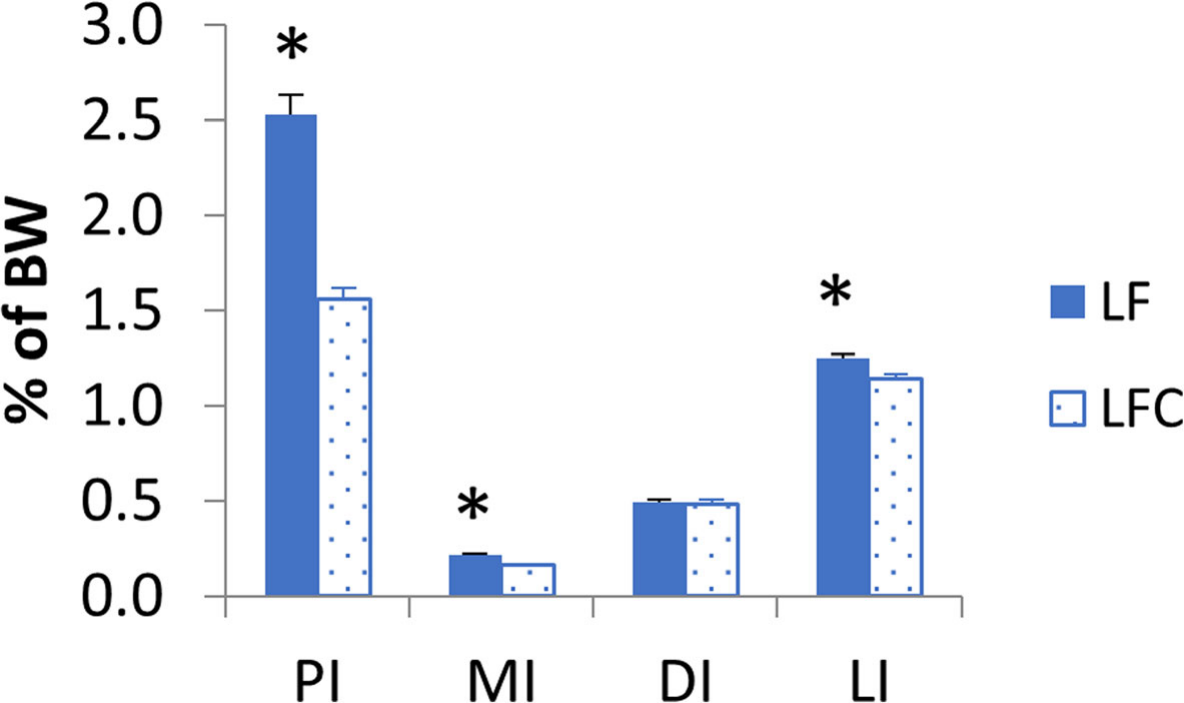
Organ somatic indices of the intestinal sections, pyloric intestine (PI), mid-intestine (MI), distal intestine (DI) and liver (LI). Values are means (PI *n* = 20 and MI, DI and LI *n* = 30) with standard errors represented by vertical bars. Significant differences (*p* <  0.05) between the LF and LFC group are indicated with *. The inclusion of choline resulted in a significant lower organ somatic index for PI, MI and LI (*p* <  0.05)

**Fig. 2:**
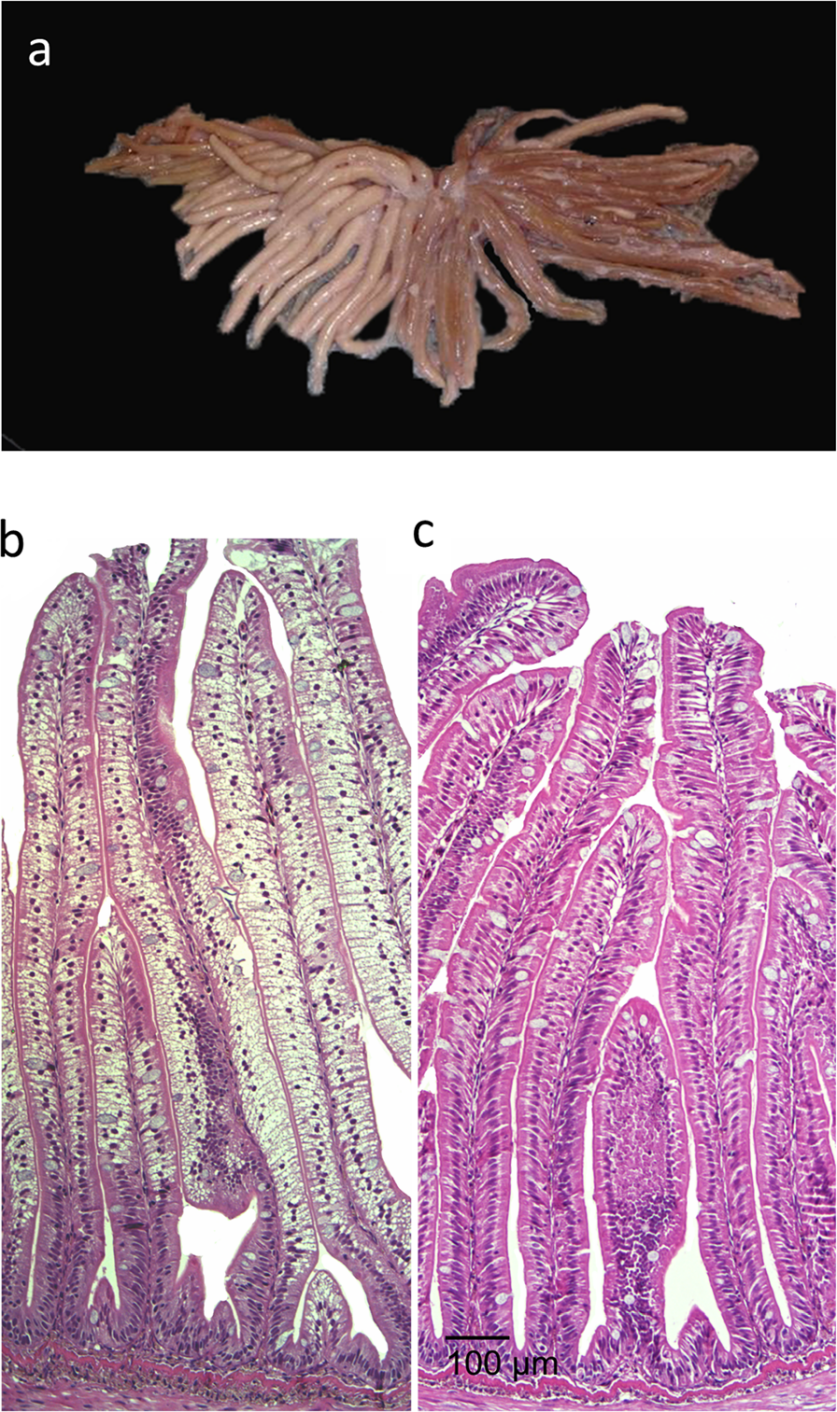
**a** example of white pyloric caeca with grossly visible of lipid accumulation. Image credit: Vegard Denstadli. Histological appearance of pyloric caeca in fish fed (**b**) the low fishmeal diet, LF and (**c**) the choline supplemented diet, LFC. Scale bare = 100 μm

**Fig. 3:**
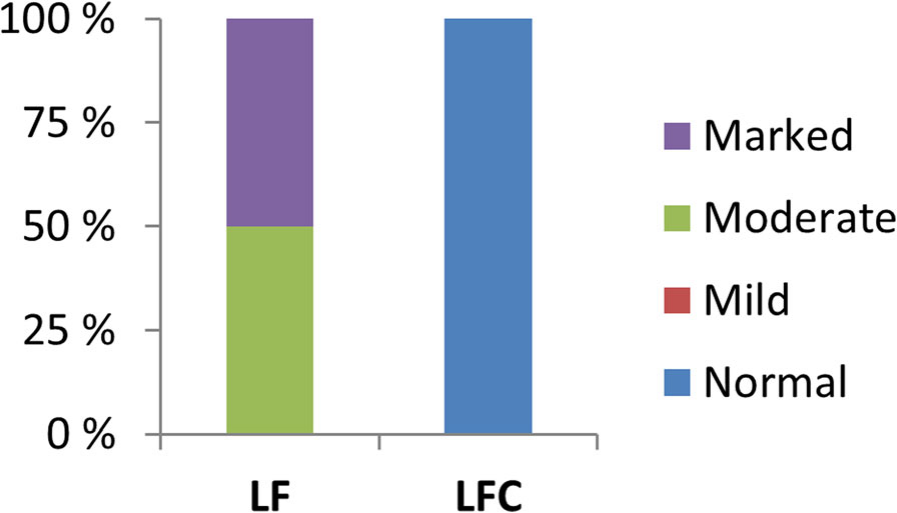
Contingency charts of the pyloric intestine showing proportions of sampled individuals that scored vacuolation grade “normal”, “moderate” and “marked” (none scored “mild”). Fish fed the low fishmeal diet displayed hyper-vacuolated enterocytes. Choline inclusion resulted in normal epithelium. The differences between the diets were significant (*p* <  0.05; Chi-squared test)

**Fig. 4:**
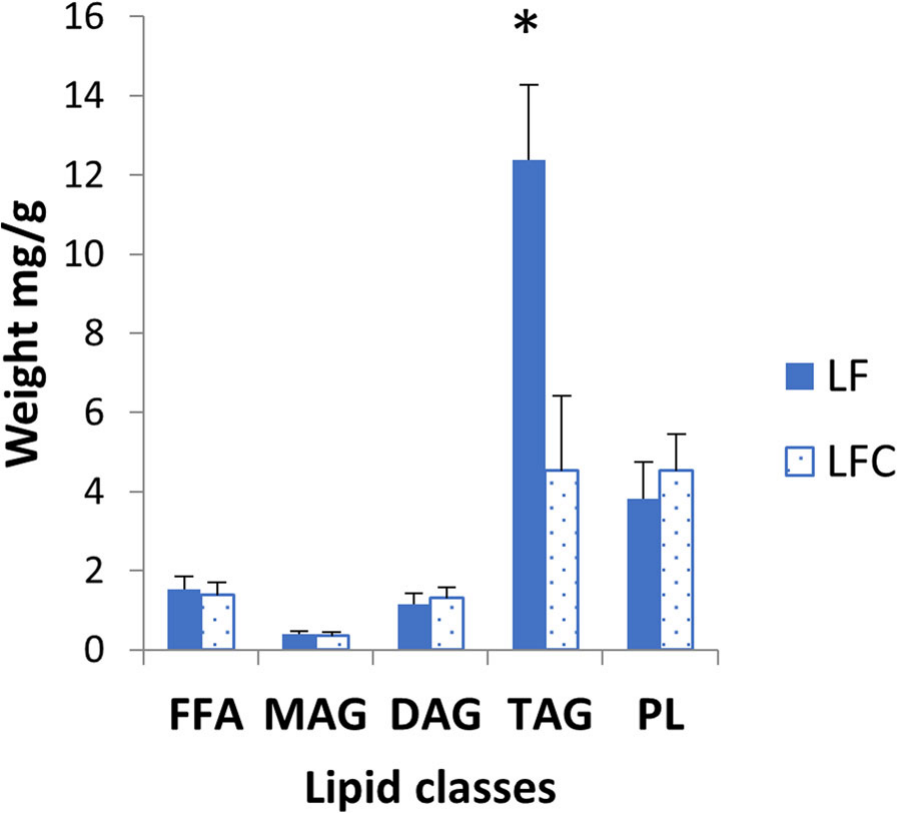
Distribution of the lipid classes; free fatty acids (FFA), monoacylglycerol (MAG), diacylglycerol (DAG), triacylglycerol (TAG) and phospholipid (PL) in pyloric caeca tissue. Values are means (*n* = 10) with standard errors represented by vertical bars. Significant differences (*p* <  0.05) between the LF and LFC group are indicated with *. The inclusion of choline resulted in a significant lower content of TAG (*p* <  0.05; T-test)

Relative organ weights of the PI, MI, DI and liver (LI) are shown in Fig. 1. Choline supplementation reduced relative weights of PI, MI and LI significantly, but not of DI. Macroscopic observations revealed white and swollen pyloric caeca in most of the sampled individuals fed the LF diet, whereas this observation was not recorded for any of the fish fed the LFC diet (Fig. 2a). Accordingly, the histological examination showed a significantly higher degree of lipid droplet accumulation in the pyloric caeca in fish fed the LF diet compared to those fed the LFC diet (Fig. 2b and c, respectively, *p* <  0.001). The degree of vacuolation of the enterocytes was 0% in sampled fish fed the LFC diet compared to 100% in the LF fed group (Fig. 3). Choline supplementation significantly lowered triacylglycerol (TAG) concentration in the tissue of the PI (Fig. 4, *p* = 0.024). No significant differences due to supplementation were found for free fatty acids (FFA), monoacylglycerol (MAG), diacylglycerol (DAG) and phospholipid (PL). The histological examination of LI vacuolation did not indicate similar effects of choline supplementation as in the pyloric caeca. No significant differences in the degree of liver vacuolation was found between the two diets (*p* = 0.867). Likewise, calculation of absolute amount of liver lipid (g) did not reveal significant differences (*p* = 0.867) between LFC and LF fed fish, 0.33 (± 0.04) and 0.33 (± 0.03), respectively.

### Intestinal gene expression

**Fig. 5:**
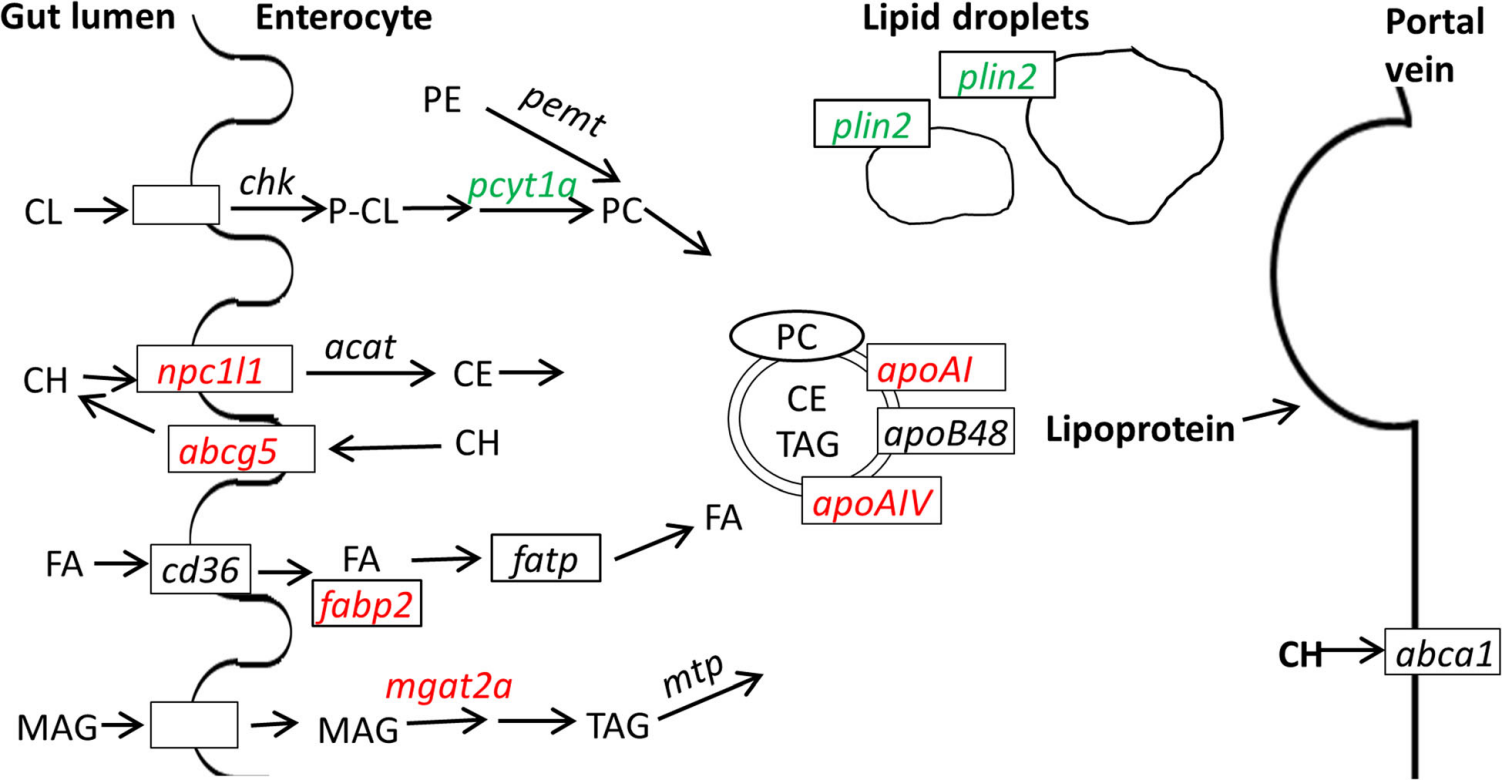
Overview of genes involved in lipid digestion and absorption in the intestine of Atlantic salmon and studied in the present study. Arrows indicates steps in the pathways. Studied genes are italicized. Green color indicates genes which were significantly down-regulated and red color indicate up-regulated genes. No color represents genes not significantly affected. Dietary choline (CL) is synthesized by c*holine kinase* (*chk*) to phosphocholine (P-CL) and after an intermediate step not studied here, *choline-phosphate cytidylyltransferase* (*pcyt1a*) to phosphatidylcholine (PC). PC could also be synthesized from endogenous phosphatidylethanolamine (PE) by *phosphatidylethanolamine N-methyltransferase* (*pemt*). PC is an important element in the membrane portion of lipoproteins preventing triacylglycerol (TAG) from leaking out. Cholesterol (CH) is transported from the lumen and over the membrane by *Niemann-Pick C1-Like1* (*npc1l1)*. *Acyl-CoA cholesterol acyltransferase* (*acat*) located in ER, facilitates the esterification of CH to cholesterol esters (CE). *ATP-binding cassette G5* (*abcg5*) returns some of the free cholesterol back to the gut for reuse. Some of the free cholesterol is also shuttled to the basolateral membrane for biogenesis of high-density lipoprotein (HDL) mediated by *ATP-binding cassette A1* (*abca1*). Fatty acids (FA) are transported from the gut lumen over the brush border membrane and into the epithelial cell by *cd36* (*cluster of differentiation 36*). The *fatty acid-binding protein 2* (*fabp2)* shuttles the fatty acids within the epithelial cell and the *fatty acid transport protein (fatp)* further to the smooth endoplasmic reticulum (ER). Monoacylglycerol (MAG) is esterified by *monoacylglycerol acyltransferase* (*mgat2a*), located in ER, to diacylglycerol (DAG) which is further transformed into triacylglycerol (TAG), a step not studied here. *Microsomal triglyceride transfer protein* (*mtp)* further facilitates the transport of TAG by assisting in the assembly of the lipoprotein. The three apolipoproteins *apoB48*, *apoAI* and *apoAIV* are important elements for successful production and secretion of the lipoprotein. The formation of lipoproteins is again an essential step for export of lipid to the general circulation and to other organs such as the liver. Excess lipid is stored as lipid droplets in the enterocytes. The lipid droplet structure and formation are regulated by the amphiphilic structural protein, *adipophilin/perilipin 2* (*plin2*)

Figure 5 illustrates the molecular regulations of the studied genes involved in synthesis of phosphatidylcholine, cholesterol, and lipids, as well as intestinal lipid transport, lipoprotein assembly and secretion. Table 4 presents the results of the effect of choline supplementation on intestinal gene expression. Expression of genes encoding three enzymes involved in the pathway of phosphatidylcholine biosynthesis was analysed. The expression of the *pcyt1a* gene was significantly down-regulated whereas the effect for *chk* showed a trend towards down-regulation (*p* = 0.068). No significant effect was observed on expression of *pemt.* Genes involved in cholesterol (CH) transport were also significantly affected in fish fed the choline enriched diet. *Niemann-Pick C1 like 1* (*npc1l1)* and *abcg5* were up-regulated. Expression of *fabp2* homologs, encoding fatty acid transporters, and the transcription factors *pparα* and *pparγ* were significantly enhanced. Also *mgat2a*, responsible for TAG re-esterification, was significantly up-regulated. A similar up-regulation was seen for both *apoAI and apoAIV*, involved in lipoprotein assembly. The general marker for lipid load of non-adipogenic cells, *adipophilin/ perilipin 2* (*plin2)* was down-regulated. The taurine transporter *slc6a6* was up-regulated in the choline treated fish.

**Table 4:** Gene expression profiling of pyloric caeca samples by qPCR

Gen category and function	Gen symbol	Fold change^a^	*P*-value^b^
Lipid uptake and transport			
Fatty acid transporter	*cd36*	1.13	0.175
Fatty acid transporter	*fabp2b*	1.37	**0.004**
Fatty acid transporter	*fabp2a1*	1.11	0.302
Fatty acid transporter	*fabp2a2*	1.01	0.962
Fatty acid transporter	*fatp*	0.89	0.120
Lipoprotein assembly	*mtp*	1.12	0.259
Lipoprotein component	*apoB48*	1.07	0.730
Lipoprotein component	*apoAIV*	1.58	**0.028**
Lipoprotein component	*apoAI*	1.42	**0.001**
Lipid droplet component	*plin2*	0.273	**< 0.001**
Nuclear receptor – regular of lipid metabolism	*pparα*	1.52	**0.037**
Nuclear receptor – regular of lipid metabolism	*pparγ*	1.38	**0.024**
Resynthesis of triacylglycerols	*mgat2a*	1.39	**0.023**
Phosphatidylcholine synthesis			
Choline transporter	*slc44a2*	1.09	0.329
Phosphatidylcholine biosynthesis	*pemt*	0.95	0.486
Phosphatidylcholine biosynthesis	*chk*	0.61	*0.068*
Phosphatidylcholine biosynthesis	*pcyt1a*	0.58	**0.004**
Cholesterol metabolism			
Bile acid nuclear receptor	*fxr*	1.15	0.415
Cholesterol biosynthesis	*hmgcr*	0.91	0.260
Cholesterol efflux transporter	*abca1*	0.95	0.650
Cholesterol efflux transporter	*abcg5*	1.61	**0.004**
Cholesterol esterification	*acat*	0.95	0.677
Cholesterol transporter	*npc1l1*	1.58	**< 0.001**
Nuclear receptor - regular of lipid and sterol metabolism	*lxr*	1.06	*0.580*
Nuclear receptor - regular of lipid and sterol metabolism	*srebp1*	0.55	0.159
Nuclear receptor - regular of lipid and sterol metabolism	*srebp2*	1.04	0.907
Taurine transporter - bile salt metabolism	*slc6a6*	1.39	**0.001**

^a^Values are mean fold change observed in the choline diet fed group in comparison with those in the control group

^b^*P*-values in bold indicate significant differences between the two treatments; italicized values represent trends

### Hepatic gene expression

In the microarray analysis (fold change > 1.6, *p* <  0.05), 168 entities were found to be differentially expressed between the two diet groups (Additional file 2). The differentially expressed genes appeared to be distributed among many functional classes, and a search for enriched GO and KEGG terms provided little meaningful information (data not shown). Among the highest responding transcripts, two innate immunity-related lectins (*nattectin*, *c-type mbl-2 protein*) were markedly induced by the choline treatment. In contrast, *rxr*, *pmm* and *sod3* were down-regulated by choline supplementation. *Perilipin 2* (*plin2)* showed up-regulation in the liver in contrast to the down-regulation found in PI. To further verify the microarray data, *rxr*, *pmm*, *sod3* and *plin2* were quantified by qPCR (Table 5). In accordance with microarray data, *pmm* and *plin2* were down- and up-regulated, respectively, whereas no differences for *rxr* and *sod3* were observed with qPCR. The lipoprotein and sterol associated transcripts measured in pyloric caeca were also quantified in liver using qPCR (Table 5). In accordance with the microarray data, we observed no significant changes for any of these transcripts. Altogether, microarray and qPCR data were closely correlated (Person’s correlation coefficient: 0.74, *p* = 0.0003).

**Table 5:** Gene expression profiling of liver samples by qPCR

Gen category and function	Gen symbol	Fold change^a^	*P*-value^b^
Lipid uptake and transport			
Fatty acid transporter	*cd36*	0.948	0.711
Fatty acid transporter	*fatp*	0.992	0.923
Lipoprotein component	*apo(B100)*_*liver*_	0.996	0.983
Lipid droplet component	*plin2*	1.626	**0.013**
Nuclear receptor – regular of lipid metabolism	*pparα*	0.660	0.182
Nuclear receptor – regular of lipid metabolism	*pparγ*	0.973	0.834
Phosphatidylcholine synthesis			
Phosphatidylcholine biosynthesis	*pemt*	0.998	0.988
Phosphatidylcholine biosynthesis	*chk*	0.505	0.116
Phosphatidylcholine biosynthesis	*pcyt1a*	0.923	0.597
Cholesterol metabolism	*abc1a1*	0.851	0.310
Bile acid nuclear receptor	*fxr*	1.154	0.265
Cholesterol biosynthesis	*hmgcr*	0.936	0.479
Cholesterol biosynthesis	*cyp7a1*	0.925	0.580
Cholesterol efflux transporter	*abca1*	0.855	0.289
Cholesterol efflux transporter			
Cholesterol efflux transporter	*abcg5*	1.063	0.601
Cholesterol transporter	*npc1l1*	0.955	0.776
Nuclear receptor - regular of lipid and sterol metabolism	*lxr*	0.837	0.156
Nuclear receptor - regular of lipid and sterol metabolism	*srebp1*	0.944	0.851
Nuclear receptor - regular of lipid and sterol metabolism	*srebp2*	1.174	0.405
ROS metabolism / antioxidant			
Superoxide dismutation	*sod3*	0.739	0.374
Nuclear receptor – control of gene transcription			
Transcription factor	*rxr*	0.789	0.104
Mannose metabolism			
Glycosylation	*pmm*	0.759	**0.029**

^a^Values are mean fold change observed in the choline diet fed group in comparison with those in the control group

^b^*P*-values in bold indicate significant differences between the two treatments; italicized values represent trends

### Blood plasma endpoints

Most of both the TAG and cholesterol in plasma was present in the high-density lipoprotein (HDL) fraction independent of treatment and the distribution of TAG and cholesterol among the lipoproteins were similar. Choline supplementation significantly decreased the plasma level of TAG reflecting reductions in HDL and low-densitylipoprotein (LDL) (Table 6). The opposite effect was seen on plasma cholesterol reflecting cholesterol increase in all the lipoprotein fractions. Plasma lathosterol, indicative of the rate of cholesterol synthesis, increased upon choline supplementation. The level of 7α-hydroxycholesterol, a metabolite in cholesterol catabolism and conversion to bile acids, also increased, whereas C4 (7α-Hydroxy-4-cholesten-3-one), a later metabolite in the cholesterol catabolism, was not significantly affected. Plasma levels of other catabolic products of cholesterol, i.e. the oxysterols 7β- hydroxycholesterol, 7β-keto-hydroxycholesterol, 24-hydroxycholesterol and 27-hydroxycholesterol were increased by dietary choline supplementation.

**Table 6:** Blood plasma variables

	LF^a^	LFC^b^	Pooled SEM	*P*-value^c^
Glucose (mmol/L) ^d^	5.3	5.9	0.22	**< 0.001**
Free Fatty Acids (mmol/L) ^d^	0.27	0.25	0.02	0.35
*Lipoptoteins*				
Total CH (mmol/L) ^d^	8.3	11.1	1.74	**< 0.001**
HDL-CH^e^	7.5	8.9		
LDL-CH^e^	1.3	1.5		
VLDL-CH^e^	0.1	0.3		
Total TAG (mmol/L) ^d^	3.3	2.5	0.27	**0.01**
HDL-TAG^e^	3.2	2.4		
LDL-TAG^e^	1	0.7		
VLDL-TAG^e^	0.5	0.8		
Bile salts (μmol/l) ^d^	20	19	8.47	0.822
Sitosterol (μg/ml) ^*f*^	71	61	6.98	0.204
Campesterol (μg/ml) ^*f*^	188	224	27.4	0.342
Lathosterol (μg/ml) ^*f*^	3.8	9.2	0.48	**< 0.001**
C4 (μg/ml) ^*f*^	0.01	0.01	0.02	0.921
*Oxysterols (ng/ml)* ^e^				
7α-hydroxy-CH	130	295		
7β-hydroxy-CH	37	139		
7-keto-hydroxy-CH	101	538		
24-hydroxy-CH	2.2	4		
25-hydroxy-CH	5	5		
27-hydroxy-CH	21	33		

^a^Low fishmeal diet

^b^Choline supplemented low fishmeal diet

^c^*P*-values in bold indicate significant differences between the two treatments; italicized values represent trends

^d^Measured for n = 20 per diet

^*e*^Lipoprotein and oxysterol profiles were measured in pooled samples of *n* = 5 per diet

^f^Measured for n = 10 per diet. Mean values with their standard errors

## Discussion

In brief, the present study revealed that choline supplementation to a plant based diet, 4.3 g/kg, improved growth by 18%, without effects on macronutrient digestibilities or other observed indicators of digestive function. The relative weight of the pyloric caeca decreased by 40% - reflecting a reduction in TAG and was shown histologically as elimination of enterocyte hyper-vacuolation. On the molecular level the supplementation caused down-regulation of genes involved in the CDP-choline pathway in which phosphatidylcholine is synthesized from free choline and a phosphorylated diglyceride (*chk* and *pcyt1a*), but had no significant effect on expression of *pemt* involved in synthesis of phosphatidylcholine from phosphatidylethanolamine via the PEMT pathway, the second pathway for phosphatidylcholine synthesis. Choline supplementation up-regulated two genes involved in cholesterol transport (*abcg5* and *npc1l1*), as well as genes involved in lipid metabolism and transport (*mgat2a* and *fabp2*), and lipoprotein formation (*apoA1* and *apoAIV*). The reduced intracellular lipid level was reflected in marked suppression of the lipid droplet marker *plin2*.

The aim of the present study was to elucidate if choline deficiency is a key contributor for LMS, and whether dietary supplementation with choline might prevent development of LMS. Our results clearly affirm these hypotheses. In this respect, our results are in line with the observations of lipid accumulation in Japanese eel fed choline deficient diets [33]. Our observations also highlight the importance of choline in lipid turnover in post-smolt Atlantic salmon, and supply information relevant for later developmental stages.

### Choline effects on performance

Choline supplementation of the feed for Atlantic salmon of the size used in the present study, start weight 362 g and final weight 740 g, was found to have a great improving effect on SGR, by 18%. Similar improvements have been observed at juveniles stages in Atlantic salmon as well as in other species [32, 34–38]. Several studies have also confirmed the requirement for phospholipid in both freshwater and marine juveniles [31, 34, 39, 40]. The 18% increase in growth rate in fish fed the choline supplemented diet may give great expectations for improvement of efficiency in production of Atlantic salmon. It should, however, be kept in mind, that the SEM indicates that the true difference might be much less, or much higher. Follow-up studies are therefore needed, to find whether similar improvements can be expected in the long run.

### Effects of choline on lipid accumulation in the pyloric intestinal tissue

There is a general understanding that TAG is the primary lipid class in lipid stores [40] and an increased supply of fatty acids promotes TAG synthesis and storage in fat cells where lipid droplets increase in abundance and size [41]. The high TAG levels and the corresponding occurrence of large lipid vacuoles observed in the pyloric caeca of the control fish suffering from LMS in the present study are in line with this. The absence of lipid droplets in pyloric caeca in choline fed fish might also be explained by phosphatidylcholine playing an important role in lipoprotein formation, and therefore in the transport of lipids across cell membranes and an efficient transport of dietary lipids from the pyloric caeca [22, 42–44]. The relatively low TAG level observed in fish fed the choline supplemented feed could also be a result of phosphatidylcholine also acting as a surfactant stabilizing growing lipid droplets and further preventing lipid droplet coalescence [41]. The concomitant alterations in expression of genes involved in phosphatidylcholine synthesis, cholesterol synthesis, lipid droplet formation, lipid transport, and lipoprotein formation and metabolism tested in the present study confirmed the importance of choline in this respect.

The cytidine (CDP)-choline pathway is the main pathway for phosphatidylcholine synthesis from dietary choline [29]. Choline kinase (*chk*), catalyzing the initial and committing step, showed a tendency to be down-regulated by choline supplementation, whereas significant down-regulation was found for *pcyt1a,* regulating the second and rate-limiting step in the CDP-pathway [28, 29]. These results are in agreement with findings presented earlier [8] showing lower expression of *chk* and *pcyt1a* in the pyloric caeca of fish fed a high fishmeal diet, supposedly with a higher choline level, compared to the expression in hyper-vacuolated pyloric caeca of fish fed a plant meal based diet with a lower choline level. The down-regulation of *chk* and *pcyt1a*, as a result of choline supplementation in the present study, could be an indication that the fish received more than enough choline and that the phosphatidylcholine synthesis was regulated through a negative feed-back control. However, regulation of *pcyt1a* activity is very complicated with important post translational steps [45]. Further studies of this rate limiting enzyme are therefore needed to understand the impact of the observed effect on *pcyt1a*. Choline supplementation did not, however, alter the expression of *pemt* in the present study, which is in agreement with previous studies carried out with mammals which showed that *pemt* is expressed mainly in the liver [29].

Choline induced the expression of both *npc1l1*, involved in the absorption of cholesterol from the intestinal lumen into the enterocytes [46] and *abcg5,* catalyzing the transport of a proportion of the free cholesterol back to the gut for reuse [47]. As such, choline seemed to promote the circulation and reuse of free cholesterol, also indicated by the increased blood plasma CH levels in choline fed fish.

Choline supplementation seemed not to influence the transport of fatty acids across the brush border membrane from gut lumen to the enterocytes and further to ER as no significant alteration of *cd36* and *fatp* expressions were observed. On the other hand, choline seemed to influence the transport of fatty acids within the epithelial cell due to the induced expression of *fabp2* in the choline fed group [48, 49]. The up-regulation of *mgat2a* indicates that choline is also important in the synthesis of MAG to DAG, which is an important intermediate for the synthesis of both TAG and phosphatidylcholine [50]. The synthesized TAG is exported from the cells in lipoproteins. Both apoAI and apoAIV are major proteins in enterocyte lipoprotein assembly [51] and were up-regulated with choline supplementation. These results support our hypothesis regarding the importance and key roles of choline for efficient lipid supply and metabolism in salmon and strengthens the suggestion that choline is important for the synthesis and secretion of lipoproteins [10, 22, 27, 42, 52]. A study on rats [53] observed an increased intestinal lipid content and an impaired chylomicron secretion as a result of choline deficiency. These observations support our findings regarding the importance of choline for proper lipid metabolism.

Another important observation was the decreased expression of *plin2,* a general marker for the lipid load of non-adipogenic cells [54]. In humans, *plin2* has been suggested as a marker for detection of lipid droplets in tissues, which further are associated with various diseases such as hepatocyte steatosis [55]. Plin2 has also been reported to coat cytoplasmic lipid droplets in enterocytes of chronic high-fat fed mice [54]. The apparent correlation between *plin2* expression and degree of enterocyte hyper-vacuolation observed in the current and previous studies [56], suggest that *plin2* may serve as a marker for intestinal lipid accumulation and steatosis in fish.

### Effects of choline on liver

The choline fed fish had significantly lower hepatosomatic index than the control, but this was not reflected in lower content of lipid, nor in histological apparent vacuolation. Both diets resulted in relatively high degree of lipid accumulation. This is in accordance with previous observations in gibel carp [32] and red drum [57] showing that dietary choline deficiency did not cause an increased accumulation of liver lipid. On the other hand, studies on common carp [58], lake trout [30], rainbow trout [31] and blunt snout bream [59] reported fatty livers in fish fed choline deficient diets. In the present study, choline supplementation caused only minor effects on the hepatic transcriptome and no genes related to lipid metabolism showed altered expression. Collectively, the lack of response to choline supplementation in liver is in sharp contrast to the marked changes observed in the intestine and clearly points towards a focus on intestinal responses in future studies of lipid accumulation and choline requirements in salmon.

### Choline effects on plasma indicators

Very low-density lipoprotein (VLDL) synthesis and assembly is regulated by the availability of triglycerides [60–63] and it seems from the tendency of the enhanced amount of both VLDL-TAG and VLDL-CH observed in the choline group that choline increased the VLDL synthesis and assembly. Even though an increase in VLDL-TAG was observed did the total level of TAG decrease in plasma in fish fed the choline enriched diet. The reduction was a result of reduced TAG in both HDL and LDL which could indicate that the lipids were successfully extracted from VLDL in the peripheral tissues [64]. A similar decrease in TAG level in plasma has been observed for juvenile lobsters [65] and cobia larvae [39] fed soy lecithin. Niu et al. [39] further suggested that this was a result of a positive effect of phospholipids on lipoprotein lipase and hepatic lipase activities for TAG uptake in liver and further distribution to other tissues. Choline also seemed to increase HDL’s, in addition to VLDL and LDL’s, capacity to bind and transport cholesterol due to the higher cholesterol amount. The present study further supports previous observations [66–68] showing that HDL is the most abundant lipoprotein carrying the main load of both cholesterol and TAG. The increase of cholesterol bound to HDL in the choline supplemented group could be a result of higher levels of phospholipids incorporated into the HDL, which in a study with rat, was shown to play a key role in modulating cholesterol efflux (transport and re-use of cholesterol) [69]. Phospholipid levels in the lipoproteins were not analysed in the present study, so this should be investigated in further studies.

## Conclusion

Choline is an essential nutrient for Atlantic salmon, even after early developmental stages. Plant based diets must be supplemented with choline to ensure normal uptake, metabolism, and export of lipids across the intestinal mucosa.

## Methods

### Diets

A low fishmeal, high plant diet (LF) was used as a reference diet, containing 10% of a 50/50 mix of Nordic LT fishmeal from the North Atlantic and Super Prime fishmeal from Peru. The total lipid content was 70% rape seed oil and 30% fish oil. The choline supplemented diet (LFC) was made by supplementing the LF diet with 4 g/kg of choline chloride. The diets contained approximately the same amount of methionine and cysteine. Table 1 shows diet formulation and analysed chemical composition. Both diets were supplemented with standard vitamin and mineral premixes in accordance with NRC guidelines (2011) and BioMar standards to meet requirements. Yttrium oxide (0.5 g/kg) was added as inert marker for estimation of nutrient apparent digestibility. The two experimental diets were produced by extrusion (feed pellet size 6 mm) at BioMar Feed Technology Centre (Brande, Denmark) using a BC 45 twin screw extruder (Clextral, France).

### Experimental animals and conditions

Atlantic salmon (*Salmo salar* L., post smolt, Sunndalsøra breed) with mean initial weight of 362 g ± 95 (mean ± SD) were pit tagged, weighed individually, and randomly allocated into four fiberglass tanks with 270 l of saltwater, two replicate tanks per diet, 35 fish in each. Each tank was supplied with flow through seawater. Salinity ranged between 32 and 33 g/l. The water flow was increased accordingly to the increase in biomass and to maintain oxygen saturation at any time above 80%. The oxygen content of the outlet water was monitored once a week or more often in periods with larger temperature variations. Temperature varied between 7.0 and 14.5 °C during the experimental period (from July to September), with an average of 9.4 °C. A 24 h light regime was employed during the experimental period. The fish were fed continuously using disc feeders aiming at an excess feeding of 15% during the trial period. Equipment for recording feed waste and hence feed intake was not available for the present experiment.

### Sampling

After 79 days, feeding was terminated. Weight and length were recorded for all fish. From each tank ten fish were anaesthetized with tricaine methane-sulfonate (MS-222). Blood was sampled from the caudal vein in vacutainers with lithium heparin. The vacutainers were stored on ice until plasma preparation. Plasma, 2 mL aliquots, was frozen in liquid nitrogen and stored at − 80 °C. Following blood sampling the fish were killed by a sharp blow to the head and opened ventrally. The gastro-intestinal tract was removed from the abdominal cavity, cleared of other organs and adipose tissue, and sectioned as follows. Pyloric intestine (PI): the section from the pyloric sphincter to the most distal pyloric caeca; mid intestine (MI): from the distal end of PI and proximal to the increase in intestinal diameter; distal intestine (DI): from the distal end of MI to the anus. The intestinal wall tissue of PI and DI was collected and weighed, whereas the digesta from these two sections were each split into two samples, i.e. the proximal half (PI1 and DI1, respectively) and distal half (PI2 and DI2, respectively). The intestinal samples were snap frozen in liquid nitrogen and stored at − 80 °C. The liver (LI) was also sampled and weighed. Another five fish per tank were euthanized and killed for sampling of LI and PI for histological and gene expression analyses. The 20 fish remaining in each tank were stripped for faeces as described by Austreng [70]. They were then fed for one more week for an additional stripping. The fecal samples were pooled for each tank, frozen immediately after stripping (N_2_) and stored at − 80 °C until analysis. Tissues sampled for histological examination were fixed in 10% neutral buffered formalin (4% formaldehyde). Samples for gene expression analyses were rinsed in sterile saline water, submerged in RNAlater®, incubated at 4 °C for 24 h and subsequently stored at − 40 °C until analysis.

### Histology

**Fig. 6:**
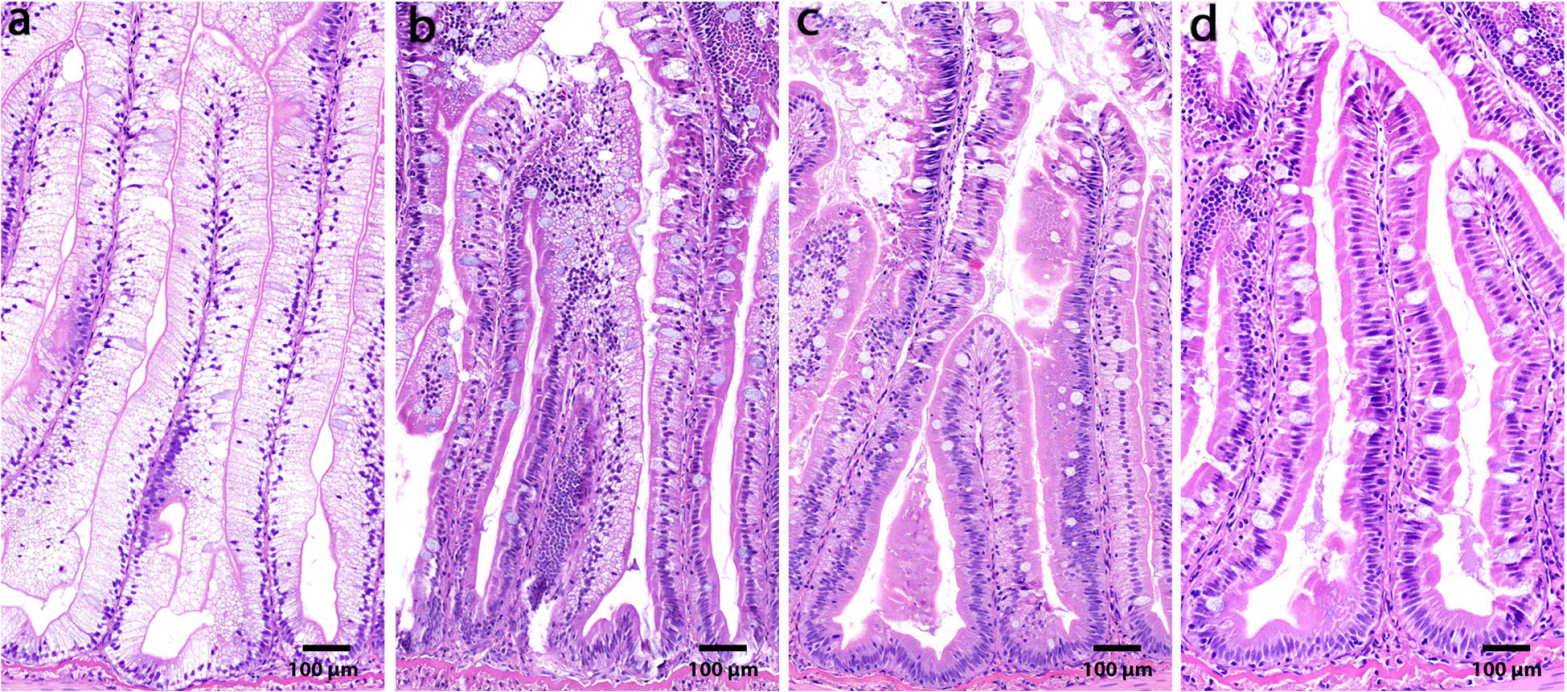
Severity of vacuolation (steatosis) of the pyloric caeca tissue, representative for (**a**) marked (**b**) moderate (**c**) mild and (**d**) normal. Scale bar = 100 μm

Pyloric caeca and liver samples were processed at the Norwegian University of Life Sciences (NMBU) using standard histological techniques: dehydration in ethanol, clearing in xylene, and embedding in paraffin before sectioning (5 μm). Hematoxylin and eosin were used for tissue staining. The samples were evaluated for enterocyte vacuolation blinded in a randomized order using a light microscope. Vacuolation was assessed based on appearance of lipid-like vacuoles, swelling and irregularity of the cells, and condensation of the nuclei. Vacuolation was assessed semi-quantitatively as the proportion of total tissue affected: normal (≤ 10%), mild (10–25%), moderate (25–50%) or marked (≥ 50%) and presented as percentage of vacuolated enterocytes (Fig. 6).

### RNA extraction

Total RNA was extracted from pyloric caeca samples (~ 30 mg) using a Ultraturrax homogenizer, TRIzol® reagent (Invitrogen, ThermoFisher Scientific) and chloroform according to the manufacturer’s protocol. Obtained RNA was DNase treated (TURBO™, Ambion, ThermoFisher Scientific) and purified with PureLink RNA mini kit (Invitrogen, ThermoFisher Scientific). Total RNA from liver samples (~ 30 mg) were also extracted using Trizol® /chloroform whereas the homogenization was carried out using a FastPrep-24 (MP Biomedicals) before the samples were purified with PureLink RNA mini kit including an on-column DNase treatment according to the manufacturer’s protocol. The integrity of the RNA from pyloric caeca samples were assessed by gel electrophoresis, and in addition selected samples were verified with a 2100 Bioanalyzer using a RNA Nano Chip (Agilent Technologies). All liver samples were evaluated by Bioanalyzer. RIN values for both pyloric caeca and liver samples were all > 8. RNA purity and concentrations were measured using the NanoDrop ND-1000 Spectrophotometer (NanoDrop Technologies). Total RNA was stored at − 80 °C until use.

### Microarrays

A two-colour microarray design was used for liver transcriptome profiling. Samples from five fish per treatment were labeled with fluorescent Cy3 and hybridized against a common reference sample (pool of 10 individual fish fed a fishmeal-based diet) labeled with fluorescent Cy5. Nofima’s Atlantic salmon 15 k oligonucleotide microarray SIQ-6 (GEO Omnibus GPL16555) was manufactured by Agilent Technologies (Santa Clara, CA USA). Reagents and equipment were from the same source unless indicated otherwise. RNA amplification and labelling were performed with a Two-Colour Quick Amp Labelling Kit and Gene Expression Hybridization kit was used for fragmentation of labelled RNA. A total of 200 ng RNA was used as input for each reaction. After hybridization in an oven over night (17 h, 65 °C, 10 rpm rotation speed), arrays were washed with Gene Expression Wash Buffers 1 and 2 and scanned with a GenePix 4100A (Molecular Devices, Sunnyvale, CA, USA). GenePix Pro 6.0 was used for spot to grid alignment, assessment of spot quality, feature extraction and quantification. STARS were used to carry out the subsequent bioinformatics data analysis [71]. Low quality spots were flagged by GenePix and filtrated away before Lowess normalization of log_2_-expression ratios (ER) was performed. Genes that passes quality control in at least four samples per group were included in subsequent analyses. The differentially expressed genes (DEG) were selected by the following criteria: fold difference > 1.6 and *p* <  0.05 (T-test). Enrichment of GO and KEGG terms in the list of DEG was assessed with Yates’ corrected chi-square using all probes that passed quality control as reference. Enriched terms corresponding to at least five differentially expressed genes were selected.

### Quantitative real-time PCR (qPCR)

Quantification of hepatic gene expression by qPCR was conducted to validate the microarray results and to examine particular genes of interest in detail. qPCR was also used for quantification of genes related to lipid and sterol metabolism and transport in pyloric caeca. Assays were carried out in accordance to the MIQE standards [72]. First strand cDNA synthesis was carried out using four fish from each tank giving a total of eight fish per treatment, and Superscript III in 20 μL reactions (Invitrogen) with total RNA (0.8 μg) and oligo (dT)_20_ primers were used. Negative controls were performed in parallel by omitting RNA or enzyme. Obtained cDNA was diluted 1:10 before use and stored at − 20 °C. Quantitative PCR primers were obtained from literature or designed using Primer3web version 4.0.0 (http://bioinfo.ut.ee/primer3/). Detailed information of the primers is shown in Additional file 1. PCR reaction efficiency (E) for each gene assay was determined separately for both pyloric caeca and liver using 2-fold serial dilutions of randomly pooled cDNA. A LightCycler 480 (Roche Diagnostics) was used for DNA amplification and analysis of the expression of individual gene targets. Each 10 μl DNA amplification reaction contained 2 μl PCR-graded water, 2 μl of 1:10 diluted complementary DNA template, 5 μl of LightCycler 480 SYBR Green I Master (Roche Diagnostics) and 0.5 μl of each forward and reverse primer. Each sample was assayed in duplicate in addition to a no template control. The three-step qPCR program included an enzyme activation step at 95 °C for 5 min followed by 40 or 45 cycles (depending on the individual gene tested) of 95 °C (10 s), 58, 60 or 63 °C (10 s depending on the individual gene tested) and 72 °C (15 s). Quantification cycle (Cq) values were calculated using the second derivative method. The PCR products were evaluated by analysis of melting curve and by agarose gel electrophoresis to confirm amplification specificity. All primer pairs gave a single band pattern on the gel for the expected amplicon of interest in all reactions. For target gene normalization, *actb*, *ef1a*, *gapdh*, *rnapolII* and *rps20* were evaluated for use as reference genes by ranking relative expression levels according to their stability, as described previously [73]. For liver samples, *rnapolII* was used as normalization factor, whereas *gapdh* was used for pyloric caeca. Relative expression of target genes was calculated using the ^Δ Δ^CT method [74].

### Chemical analyses

Diets and faecal samples were analysed for dry matter (after heating at 105 °C for 16–18 h), ash (combusted at 550 °C to constant weight), crude protein (by the semi-micro-Kjeldahl method, Kjeltec-Auto System, Tecator, Höganäs, Sweden), lipid (diethylether extraction in a Fosstec analyzer (Tecator) after HCL-hydrolysis), starch (measured as glucose after hydrolysis by alpha-amylase (Novo Nordisk A/S, Bagsvaerd, Denmark) and amylo-glucosidase (Bohringer Mannheim GmbH, Mannheim, Germany), followed by glucose determination by the “Glut-Dh method” (Merck Darmstadt, Germany)), gross energy (using the Parr 1271 Bomb calorimeter, Parr, Moline, IL, USA) and yttrium (by inductivity coupled plasma (ICP) mass-spectroscopy as described by Refstie et al. [75]. The plasma variables; free (non-esterified) fatty acids, cholesterol and total triacylglycerides were analysed according to standard procedures at the Central Laboratory of the Norwegian University of Life Sciences (NMBU). Lipoprotein profile analyses (HDL, LDL and VLDL) in plasma were carried out by size exclusion chromatography and measurements of cholesterol and triglycerides on-line using microliter sample volumes as described by Parini et al. [76]. Isotope dilution mass spectrometry as described by Lund et al. [77] was used for analyzing lathosterol. 7α-hydroxy-4-cholesten-3-one (C4) was analyzed by isotope dilution and combined HPLC-MS as described by Lövgren-Sandblom et al. [78]. Plasma levels of oxysterols, sitosterol and camposterol were analyzed by isotope dilution and combined GC-MS after hydrolysis as described by Dzeletovic et al. [79] for the first mentioned and by Acimovic et al. [80] for the last two mentioned. The lipid classes free fatty acids (FFA), monoacylglycerol (MAG), diacylglycerol (DAG), triacylglycerol (TAG) and phospholipid (PL) in the pyloric caeca were extracted using the Folch procedure [81], then analysed using HPTLC Silica gel 60 F plates. DigiStore 2: documentation was used for visual documentation and the integration program WinCats was further used for calculating the amount of the lipid classes.

### Enzyme analyses

Brush-border membrane enzyme activity were analysed by measuring the activity of the enzyme leucine aminopeptidase (LAP; EC 3.4.11.1) in intestinal tissue homogenates. The homogenates were prepared from tissue thawed on ice-cold tris-mannitol buffer (1:20 w/v) containing the serine proteinase inhibitor 4-[2-Aminoethyl] benzensulfonylfluoride HCL (Pefabloc® SC; Pentapharm Limited). LAP activity was then determined colorimetrically with a kit (Sigma procedure no. 251) using L-leucine-β-napthylamide as substrate.

### Calculations

Growth of the fish was calculated as specific growth rate (percent growth per day): SGR = ((ln FBWg / ln IBWg) / D) X 100. IBW and FBW are the initial and final body weight (tank means) and D is number of feeding days. Organ somatic index was calculated as percentages of the weight of the organ in relation to body weight. Apparent digestibilities (AD) of main nutrients was estimated by using Y_2_O_3_ [82] as an inert marker and calculated as: AD_*n*_ = 100 – (100 X (M_feed_/M_faeces_) X (N_feed_/N_faeces_)), where M represents the percentage of the inert marker in feed and faeces and N represents the percentage of a nutrient in feed and faeces.

### Statistical analysis

The diets in the present study were part of a larger trial. To obtain the best estimate of variance of tank means (SEM), results from all treatments were included. The other results of the experiment are published elsewhere [66, 67]. Tank was the experimental unit for all responses except for the histological observations for which the individual fish were the unit. Statistical analyses were performed using SAS (SAS Institute Inc., Cary, NC, USA). Data was analysed using the General Linear Model procedure with diets and tanks as class variables. Specific differences were evaluated by Duncan’s test. The level of significance was set to *P* <  0.05, and *P*-values between 0.05 and 0.1 were considered as indications of effects and mentioned as trends. All data are means ± SEM. A Chi-squared test was used for analyzing histology data.

## Supplementary information

**Additional file 1.** Primer pair sequences, efficiency, amplicon size and annealing temperature for the genes used for real-time PCR.

**Additional file 2.** Differentially expressed genes between hepatic transcriptomes of fish fed the low fishmeal diet (LF) and fish fed the choline supplemented diet (LFC).

## Data Availability

The datasets used and/or analysed during the current study are available from the corresponding author on reasonable request. The datasets generated and/or analysed during the current study are available in the National Center for Biotechnology Information (NCBI) Gene Expression Omnibus NCBI repository, with accession no. GSE51887.

## Abbreviations

AD: Apparent digestibility
DAG: Diacylglycerol
DI: Distal intestine
DI1: Proximal half of distal intestine
DI2: Distal half of distal intestine
FFA: Free fatty acids
HDL: High-density lipoproteins
IBW: Initital body weight
LAP: Leucine aminopeptidase activity
LDL: Low-density lipoproteins
LF: Low fishmeal
LFC: Choline supplemented low fishmeal
LI: Liver
LMS: Lipid malabsorption syndrome
MAG: Monoacylglycerol
MI: Mid intestine
OSI: Organosomatic indices
PI: Pyloric intestine
PI1: Proximal half of pyloric intestine
PI2: Distal half of pyloric intestine
PL: Phospholipid
qPCR: Quantitative real-time polymerase chain reaction
SGR: Specific growth rate
TAG: Triacylglycerol
VLDL: Very low-density lipoproteins
